# Supervised exercise protocol for lower limbs in subjects with chronic venous disease: an evaluator-blinded, randomized clinical trial

**DOI:** 10.1186/s13063-020-04314-1

**Published:** 2020-05-19

**Authors:** Esther Fernandes Tinoco Volpe, Vanessa R. Resqueti, Ana Aline Marcelino da Silva, Lucien Peroni Gualdi, Guilherme A. F. Fregonezi

**Affiliations:** 1 PneumoCardioVascular Lab/HUOL Hospital Universitário Onofre Lopes, Empresa Brasileira de Serviços Hospitalares (EBSERRH) Departamento de Fisioterapia Universidade Federal do Rio Grande do Norte, Campus Universitário Central, Caixa Postal 1524/ Campus Universitário Lagoa Nova, Natal, Rio Grande do Norte CEP: 59078-900 Brazil; 2 Laboratório de Inovação Tecnológica em Reabilitação, Departamento de Fisioterapia, Universidade Federal do Rio Grande do Norte, Natal, Rio Grande do Norte Brazil; 3 Faculdade de Ciências da Saúde do Trairi, Universidade Federal do Rio Grande do Norte (UFRN), Santa Cruz, Rio Grande do Norte Brazil

**Keywords:** Exercise, Resistance training, Exercise therapy, Venous insufficiency, Randomized controlled trial, Rehabilitation

## Abstract

**Background:**

Chronic venous insufficiency (CVI) causes pathophysiological changes in the lower-limb muscles, particularly the calf muscles, and limits ankle range of motion (ROM). These changes reduce functional activities and decrease quality of life (QOL). Although several studies have shown the benefits of exercise (strengthening the calf muscles to improve calf-muscle pumping and QOL) in patients with CVI, few studies are randomized controlled trials. This has led to a weak indication of exercise for the treatment of patients with CVI. The aim of this study is to analyze the effects of a supervised exercise program to improve calf-muscle endurance as well as QOL in patients with CVI.

**Methods/design:**

This is an evaluator-blind, randomized clinical trial with an 8-week duration and a follow-up evaluation at week 16. A pilot study with subjects with a CVI diagnosis will be performed to calculate sample size. The participants will be randomly allocated (1:1) into a treatment or a control group (usual care/no intervention). The treatment intervention consists of a bi-weekly supervised exercise program of the lower limbs that will include aerobic training, strengthening and cardiovascular exercises. The participants from both groups will participate in a health education lecture. Primary outcomes are changes in calf-muscle endurance and QOL score. Secondary outcomes are changes in exercise capacity, ankle ROM, electrical muscle activity and cardiac output. The first statistical comparison will be performed after 8 weeks’ intervention.

**Discussion:**

Patients with CVI may have an impaired calf-muscle pump and decreased exercise capacity. A randomized controlled trial evaluating a supervised exercise program should provide much needed information on the management of CVI to promote health and independence.

**Trial registration:**

This study was registered on the Brazilian Clinical Trials Database (REBEC) (RBR-57xtk7). The results will be disseminated at scientific events, presentations, and publications in peer-reviewed journals.

## Background

Chronic venous insufficiency (CVI) is a common health problem and may cause significant morbidity and mortality [1]. It develops when the venous pressure is increased and blood return is impaired. Several mechanisms may result in blood-flow impairment including incompetent valves (superficial or deep veins), perforating veins, venous obstruction or a combination of these mechanisms. This leads to general or local venous hypertension, mainly while standing or ambulating, contributing to macro- or microcirculatory hemodynamic impairments [2] and local tissue ischemia [3]. CVI includes a wide range of clinical signs varying from varicose veins and uncomplicated telangiectasia to venous ulceration [1, 4].

Patients with CVI may develop musculoskeletal changes, mainly in the calf muscles (gastrocnemius and soleus), such as muscle-fiber atrophy [5], leading to abnormal cadence [6] and reductions in muscle strength and function [7]. A decrease in the skeletal-muscle pump worsens venous hypertension, leading to excessive accumulation of fluid and fibrinogen in subcutaneous tissue, which in turn causes edema and/or lipodermatosclerosis, and may lead to venous ulcers [2, 8, 9]. Additionally, ankle-joint movement is decreased [10] and is associated with a higher risk of venous ulcers. Conversely, satisfactory ankle dorsiflexion and effective function of the calf-muscle pump prevents edema and venous ulcers [11].

Effective calf-muscle pumping, even in the presence of valvular dysfunction or venous obstruction, may develop a compensatory mechanism (assisting in venous return) and thereby decrease CVI symptoms. Some studies have shown the benefits of exercise therapy in participants with CVI with an emphasis on strengthening the calf muscles for improved calf-muscle pumping [12–14]. An improvement in the hemodynamic function of calf-muscle pumping (represented by the ejection fraction and residual volume fraction) was described after a supervised lower-limb strengthening and stretching exercise program [13], and after eight consecutive days of isometric strengthening and resistance exercises in the calf muscles [15] for participants with CVI. Other authors [16, 17] have highlighted the importance of progressive resistance exercises and supervised aerobic training to promote ulcer healing and improve cutaneous microvascular reactivity in participants with CVI.

A systematic review [18] focusing on physical exercise for the treatment of CVI without ulcers found only two studies that met the eligibility criteria. Although both concluded that physical exercise led to an increase in venous filling time and ejection fraction, indicating an improvement in venous hemodynamics, the evidence quality was considered very low with a high risk of bias. Considering that resistance training and progressive isometric exercises are routinely prescribed for other cardiovascular diseases (such as peripheral obstructive arterial disease and coronary artery disease) [17] and that evidence-based exercise programs tested in CVI participants remain limited, we aim to assess the efficacy of a supervised exercise program to improve calf-muscle endurance and QOL as well as to asses ankle range of motion (ROM), electrical muscle activity, exercise capacity and cardiac output in participants with CVI. Additionally, we aim to determine whether the possible gains achieved in a supervised training program remain after an unsupervised period.

### Hypotheses

We hypothesize that a supervised lower-limb muscle-training program in participants with CVI will improve calf-muscle endurance and QOL scores.

## Methods/design

This is an evaluator-blinded, randomized controlled superiority trial with two parallel groups, and a 1:1 allocation ratio, with an 8-week intervention period and outcomes measured at baseline, 8 weeks and 16 weeks as a follow-up evaluation. The first statistical comparison will be performed after 8 weeks of intervention.

### Trial design

The evaluations will start in January 2020. After evaluation, participants will be randomly allocated to two evaluator-blinded groups: the treatment group (TG) and the control group (CG). The randomization.com program will be used to randomize the participants and stratification will be performed to ensure balance between the groups within two strata (Clinical, Etiology, Anatomical, Pathophysiology (CEAP) 2 and 3 and CEAP 4 to 6). A separate, blinded researcher will contact the participants by telephone to ensure allocation concealment during screening, consent and initial assessment. A study researcher responsible for implementation will apply the exercise program.

**Fig. 1:**
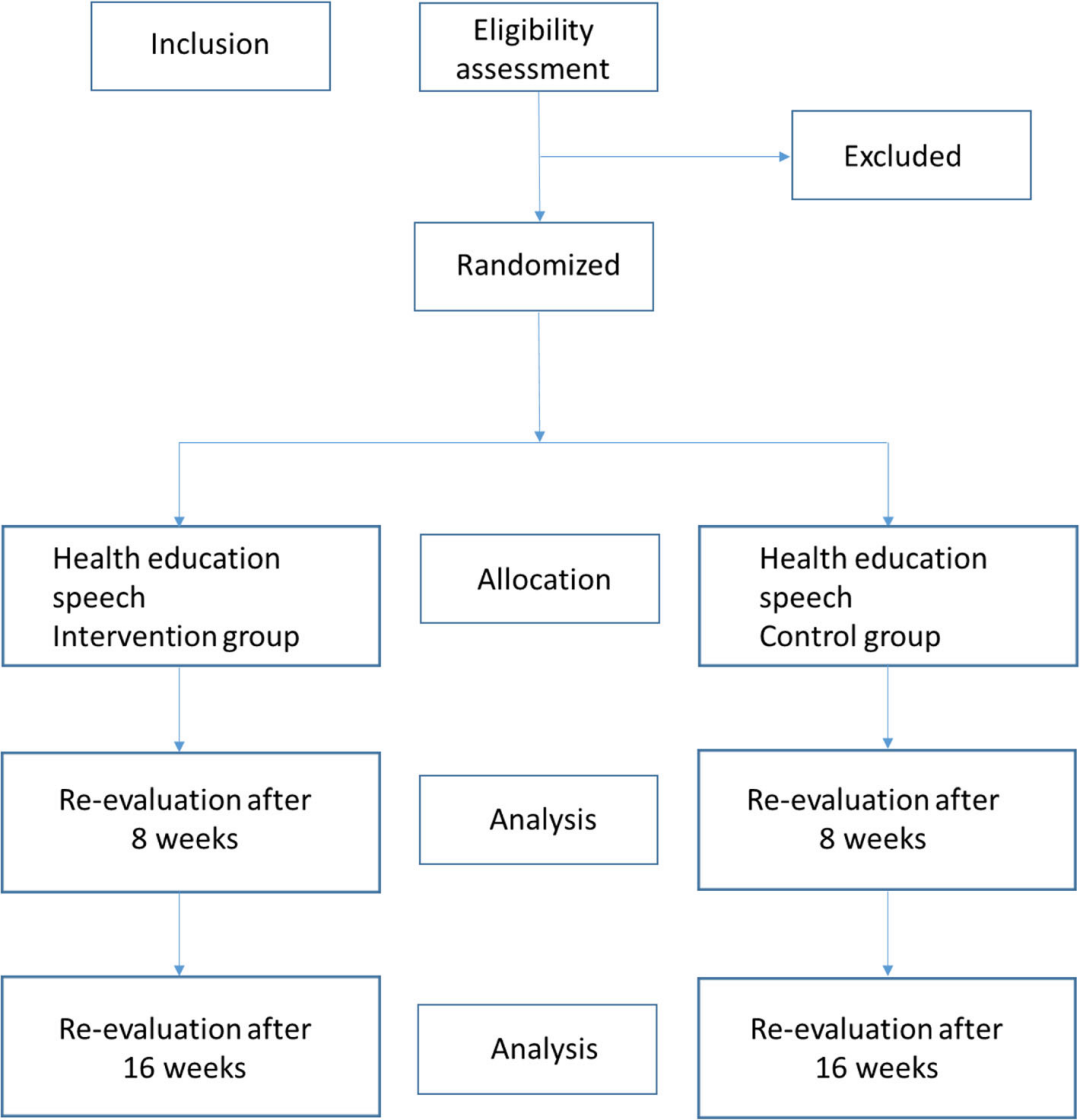
Trial design

**Fig. 2:**
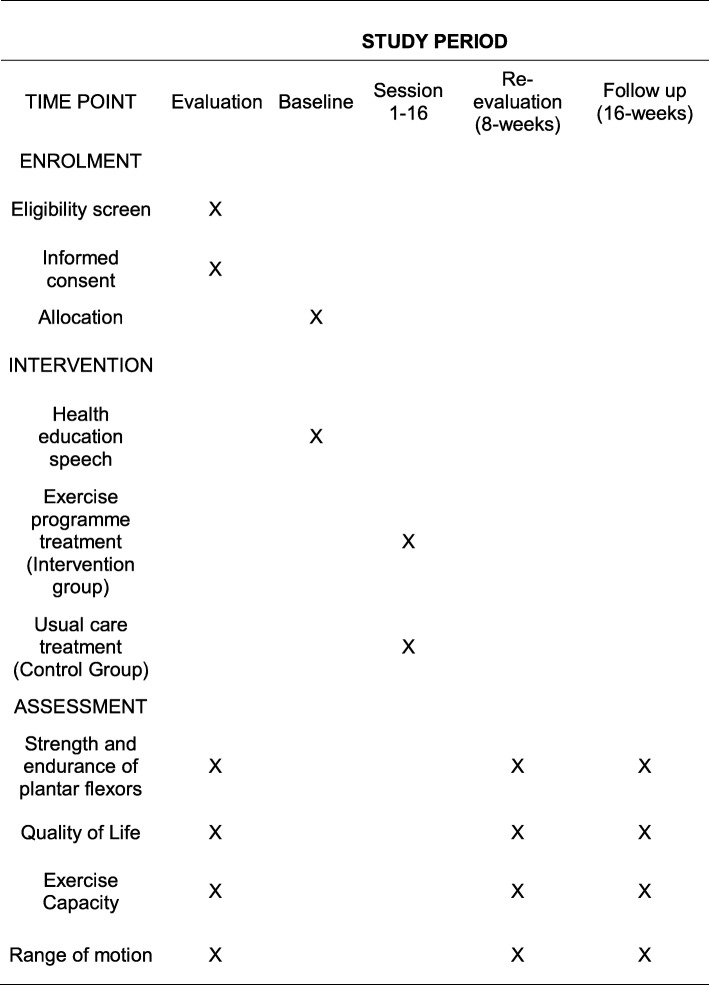
Overview of assessment. Standard Protocol Items: Recommendations for Interventional Trials (SPIRIT) Figure showing the schedule of enrollment, interventions and assessment

Patients in the TG will perform the exercise program as described in this protocol and will start 1 week after the initial evaluation. The patients in the CG will continue their usual treatments (medication use, compression stockings and medical guidance). After 8 weeks of intervention, the patients will be re-evaluated (using the same initial evaluation questionnaire) by the same blinded evaluator for each group. The primary outcomes will be calf-muscle endurance and QOL and the secondary outcomes will be exercise capacity, ROM electrical muscle activity, and cardiac output. A final reassessment will be performed 8 weeks after the re-evaluation, as shown in Figs. 1 and 2 (Standard Protocol Items: Recommendations for Interventional Trials (SPIRIT) Figure).

All participants will receive educational information regarding the disease, as well as the usual care (hygiene and compressive techniques) and treatments to improve symptoms and quality of life (QOL).

### Study setting

The evaluations will be performed at the Pneumocardiovascular Laboratory, and the intervention program will be performed at the Physical Therapy Office, both located in the University Hospital of Natal/RN, Brazil.

### Recruitment

Participants will be recruited at the Medical Clinic of the University Department of Clinical Medicine in the city of Natal/RN, Brazil. This outpatient facility has five physicians specialized in vascular surgery. The inclusion and exclusion criteria will be presented to the physicians personally. The physicians will be asked to refer all participants who meet the inclusion criteria for the study. All the participants will sign the informed consent form which will be explained by the evaluator before the evaluation.

### Participants

Potential participants with stable CVI who meet the eligibility criteria have been recruited from the Vascular Surgery Outpatient Clinic. They have been invited to participate through an informed consent process. The initial evaluation will be performed by a blinded evaluator through a questionnaire that will include socio-demographic data, co-morbidities, time of CVI diagnosis, and detailed clinical and functional information. The disease classification will be based on the CEAP criteria including: clinical manifestations (C), etiological factors (E), anatomic distribution of disease (A) and underlying pathophysiological findings (P) [4]. Ulcer morphological characteristics (if present), ROM, exercise capacity, muscle endurance and parameters related to QOL will also be assessed.

All participants will sign the informed consent form that will be explained before the evaluation. During the functional tests, hemodynamic cardiac parameters (cardiac output, ejection fraction and systolic volume) and bilateral electrical activity of the calf and tibialis anterior muscles will be assessed.

### Eligibility criteria

The inclusion criteria for this study are male and female patients aged between 35 and 69 years, with CVI diagnosed by venous vascular echo-Doppler (VED), with CEAP between 2 and 6, and without peripheral arterial disease (PAD) (ankle-brachial index (ABI) < 0.9) [19]. The exclusion criteria for this study are participants who do not agree to participate, have ulcers > 4 cm in diameter, or clinical signs and/or confirmed diagnosis of infection as well as the patients who already perform any type of self-reported supervised or unsupervised exercise program (whereas exercise is a subset of physical activity that is both regular and structured) [20], are unable to attend physiotherapy sessions twice a week, and/or have co-morbidities incompatible with moderate to intense exercises [21] such as: acute or uncontrolled congestive heart failure, uncontrolled or unstable angina, uncontrolled cardiac dysrhythmia causing hemodynamic symptoms, severe symptomatic aortic stenosis, recent deep venous thrombosis, recent pulmonary embolism, acute pericarditis or myocarditis, dissecting aneurysm (known or suspected), unstable or uncontrolled blood pressure (systolic pressure > 160 mmHg, diastolic pressure > 100 mmHg), acute systemic infection, or uncontrolled diabetes, limiting musculoskeletal diseases, or difficulty in understanding the study activities.

### Blinding

The researcher who will perform the initial and final evaluations will be blinded to the participants’ allocation group. The participants will be instructed not to make any comments regarding group allocation. The evaluator will not have access to the treatment site where the protocol will be performed to reduce the possibility of interfering with the blinding.

## Interventions

### Exercise prescription

The exercise program will consist of (1) aerobic training, (2) strengthening, and (3) unsupervised stretching exercises performed at home. The cardiovascular exercises will be performed using a cycle ergometer and the rubber step. The muscle strengthening will be performed using resistive loads for the calf muscles. The participants will receive a written and illustrated guide for performing active stretching exercises of the calf and tibialis anterior muscles once a day for 20 s (each muscle group) at home 24 h after supervision [22]. The exercise program will last ~ 40 min and will be performed twice a week, for a total of 16 sessions. Heart rate (HR) and blood pressure will be checked at the beginning and end of the training, as well as at the end of each series.

1. Aerobic training
  - Cycle ergometer exercise

The perceived fatigue of the participants will be measured using the modified 0–10 BORG scale [23], every 5 min. The participants will warm up for 5 min on the cycle ergometer without a load at the beginning of the protocol. Next, the participants will perform the cardiovascular exercises using the cycle ergometer for 15 min. The load will be adjusted at a setting up to moderate intensity (between 4 and 6 of the modified BORG scale 0–10).

b) Bench step-up exercise

The bench step-up exercise will be performed on a rubber step at a height of 20 cm. The participants will be instructed to walk up and down on steps with one foot at a time using free cadence. They will be instructed to perform the movement as fast as possible for a maximal of 12 repetitions and exercise progression (5 to 10 repetitions) will be weekly increased, according to individual tolerance.

The load during the program execution may be decreased, the rest time increased or the session interrupted if the subject reports very intense perceived fatigue (7 or above, BORG scale), complains of limiting pain, or develops symptoms incompatible with physical activity. The participants will perform only the exercises under supervision of the physiotherapist responsible for the study protocol. The patients showing exercise limitation due to pain, change in medication, undergoing any alternative treatment, or missing three consecutive intervention sessions will be excluded. The data will be included in the records for further analysis even after exclusion. Medical assistance will be provided to any participant who has an injury caused by the study in accordance with resolution 466/12 of the National Health Council.

## Resistance training

To strengthen and increase the endurance of the calf muscles, the patients will perform at 80% of 1 repetition maximum (RM). The submaximal load of 10-RM estimated percentage 80% of 1 RM [24] will be individually calculated based on momentary muscle failure (inability to perform 10 concentric contractions without significant postural change and repetition velocity during changes against a certain resistance) [25]. To calculate the submaximal load of 10 RM, weight will be added until momentary muscle failure of the individual is achieved during the calf-raise exercises. The last load successfully lifted before momentary muscle failure will be used. The calculated load will be used to customize the training level and will be changed according to the patient’s weekly performance. The exercise will consist of three sets of 10 repetitions with a 1-min rest interval. Successive load progression will be made during the program, maintaining the same volume according to the patient’s performance. The exercises during the initial sessions will be performed without any load. The loads will be applied using an adjustable weight-vest according to each patient.

### Health education speeches

All the participants will be invited to attend an educational speech about the disease, risk factors, lifestyle changes and lower-limb care (hygiene, exercises, dressings), as well as the benefits of using compressive techniques. The speeches will be performed (immediately after the first assessment) by a blinded evaluator for each allocation group who will perform the evaluations and reassessments.

### Prescription for compression stockings

Compression stockings will be prescribed for those participants who are not yet using compressive techniques. Prescription will be based on clinical severity. CEAP C2 to C3 compression of 20–30 mmHg, CEAP C4 to C6 30–40 mmHg compression and, for patients with recurring ulcers, compression of 40–50 mmHg [2]. Compliance (adherence to the use of socks) will be recorded daily in a notebook.

### Strategies to improve adherence to the intervention protocols

After the first session of the exercise program, the participants will receive a follow-up guide containing questions regarding compressive therapy, stretching and lower-limb positioning during rest.

### Conventional treatment for the control group

Conventional treatment will consist of the health education speech, use of prescribed medication, compression stocking and medical guidance. The controls will be instructed to maintain their usual activities and treatments and not perform any type of supervised exercises during the 2 months after their first evaluation.

## Outcomes

### Primary outcome

#### Assessment of calf-muscle endurance

The external cadence heel-raise test [26] adapted to the bipodal position [27] will be used to assess calf-muscle endurance. Calf-muscle endurance will be assessed by the number of repetitions achieved during the test. The participants will be instructed to remain in an orthostatic position, barefoot with bipodal support. Their balance will be maintained through contact of the fingertips of the dominant hand on a wall with elbows flexed at 90°. Next, the participants will be asked to raise their heels from the floor. The evaluator will record the maximum height reached by the participant using a stadiometer and will explain to the participants that they should achieve the marking with their heads during the heel-raise movement. The cadence of the test will be determined by a metronome (46 beats per minute) and they will be encouraged to perform as many heel-raise movements as possible. The test will be interrupted in the following situations: if the participant does not reach maximum elevation on two consecutive occasions; transfers too much weight against the wall on two consecutive occasions; performs knee flexion on two consecutive occasions; or asks to interrupt the test [28]. Blood pressure and HR will be monitored at rest, immediately after the test and after a resting period post test.

#### Quality of life (QOL)

A Brazilian version of the VEINES-QOL (Venous Insufficiency Epidemiological and Economic Study-Quality of Life) questionnaire [29] will be used to assess QOL. This instrument assesses 26 items: 10 symptom-related items, nine items regarding daily-life activities, one item related to the time of the day when the symptoms are more intense, one item regarding the changes due to the disease in the last year, and five questions about the psychological impact of the symptoms/disease. Symptoms, daily-living limitations and psychological impact questions are related to the last 4 weeks. Each domain has a different scale and will be analyzed separately.

### Secondary outcomes

#### Assessment of exercise capacity

The step test (ST) will be used to assess exercise capacity and will follow the recommendations previously published [30]. It will last for 6 min (Step test 6; ST6), and the values ​​for HR, systemic arterial pressure, dyspnea score using the modified BORG scale (0–10) and oxyhemoglobin saturation using a digital oximeter (SpO_2_%) will be registered at baseline and after the test. The number of steps will be used to analyze the participant climbing up and down (one cycle of climbing up and down was counted as one step). A 20-cm-tall rubber step will be used and the patient will be advised to wear comfortable clothing and shoes. The examiner will initially demonstrate how to perform the test. The subject should start the test using the right leg, followed by the left leg. To go down the step, the participant must follow the same order; first the right leg followed by the left leg, and then repeat the sequence at the given time. The participant will be instructed to perform the test as quickly as possible with free cadence and without discomfort. The test will be discontinued if the HR exceeds 85% of age-predicted maximal HR [31], if the participant points to a value greater than 7 on the modified BORG scale, or if the participant asks on their own initiative to finish the activity. If the participant reports fatigue or dyspnea they will be instructed to stop the test and rest on a chair. They will also be instructed to continue the test as soon as possible. During the resting period, the timer on the stopwatch will continue and the examiner should record the break. The patient will be verbally encouraged every minute without excess stimulation. The examiner will warn the subject with a clear “stop” message when 15 s are left. The same vital signs and symptom scores will be evaluated at the end of the test.

#### Ankle range of motion

The joint movement range will be measured using a simple goniometer. The measurements will be standardized for all the patients in a sitting position with knees extended and ankles initially at 90°. One arm of the goniometer will be positioned over the lateral malleolus, while the movable arm will be positioned over the fifth metatarsal accompanying the entire ankle range for dorsiflexion and flexion-extension [32].

#### Electrical activity assessment

During the tests (external cadence heel-raise test and the step test), electrical activity of the calf and tibialis anterior muscles will be assessed using superficial electromyography (SEM). The electrodes will be placed according to Surface ElectroMyoGraphy for the Non-Invasive Assessment of Muscle (SENIAM) guidelines for Surface ElectroMyoGraphy (SEMG) placement [33]. For the medial portion of the gastrocnemius muscle, the electrodes will be placed on the most prominent bulge of the muscle. For the tibialis anterior muscle, the electrodes will be placed at one third of the line between the tip of the fibula and the tip of the medial malleolus. A signal-conditioning module (TeleMyo DTS desk Receiver®, Noraxon USA Inc., Scottsdale, AZ, USA) with four wireless sensors (Clinical DTS-Noraxon®, Noraxon, Scottsdale, AZ, USA) will be used. The signals will be captured and stored using the MR 3.8 software (Noraxon USA Inc., Scottsdale, AZ, USA). The mean peak will be used to normalize the electrical signal [34], and the electromyographic signal will be analyzed at four moments (25%, 50%, 75% and 100%).

#### Cardiac output assessment

A non-invasive registering of cardiac output will be performed by a cardiograph through electrical impedance using the PhysioFlow® Q-Link equipment (Paris, France). This method has been shown to be valid and reliable at rest and during submaximal exercise in patients with normal cardiorespiratory function [35]. The skin preparation and placement of the electrodes will be according to the manufacturer’s recommendations. Following trichotomy, alcohol cleansing and abrasion with Nuprep® gel (Weaver, Aurora, CO, USA), six transcutaneous electrodes (PhysioFlow PS-50, Manatec Biomedical, Macheren, France) will be placed on the patient’s upper region. Next, two emitting electrodes will be placed on the left base of the neck, above the supraclavicular fossa and two sensing electrodes will be placed below the xiphoid process on the right side of the patient. During the functional tests, the two sensing electrodes will be positioned in the paravertebral area, at the level of the xiphoid process. One electrode will be located in the middle of the sternum and the other at the left lateral chest wall (sixth intercostal space) to conduct the electrocardiogram signal.

### Sample size

The sample size will be calculated based on a pilot study with eight patients (four patients in each group) using a multivariate analysis of variance (manova) test with repeated measures, within-between interaction with two groups and two measurements by analyzing calf endurance and the standard deviation of the number of repetitions from the pilot study. A two-tailed alpha error of 0.01 will be considered with a power of 80% considering clinical improvement for subjects with chronic venous disease after the supervised exercise protocol. The effect size will be calculated at the end of the protocol considering the number of repetitions of the calf-endurance test of all study participants. Moreover, considering a 20% loss to follow-up and 5% missing data, the number of participants will be increased by at least 20% based on sample size. The GPower (Germany) version 3.1 program will be used for statistical analysis.

### Data collection, management and analysis

#### Data collection

The baseline and revaluation data will be collected by a trained physical therapist using a protocol for the outcomes related to the questionnaire (VEINES-QOL), demographic data, and CEAP classification. For the physical tests the evaluator will perform a brief orientation, allowing the patient to practice the movement before beginning the test.

A follow-up report will be available to all participants of the IG. It will include evaluation and re-evaluation information for the next medical appointment. The evaluator will refer to the subject’s physician to identify those excluded from the study due to ankle-brachial index (ABI) values below 0.9 [19].

The data will be stored in one of the laboratory computers and double entry will be performed by two study researchers. Access to the data will be limited to the study researchers and any other access must be authorized by the coordinator.

All data collected will be available on the evaluation form and in the proper computer file. Access to these data will be limited only to researchers with prior permission from the study coordinator. The exercise program will be supervised by a physical therapist with expertise in exercise physiology and experience in supervised exercises. All data regarding the treatment protocol will be registered in the subject’s file and attached to the participants’ medical charts.

The statistical analysis will be performed using the GraphPad Prism version 5.0 statistical package software (GraphPad Software Inc., San Diego, CA, USA). The results of patient baseline characteristics and outcome variables (both primary and secondary) will be summarized using descriptive summary measures: expressed as mean (standard deviation) or median (range) for continuous variables and *n* (%) for categorical variables. The sample normality will be tested by the Shapiro-Wilk test. Treatment effects or differences between the study groups for primary and secondary outcomes will be analyzed by linear mixed model for group (usual care versus intervention) and time (baseline, 8 weeks and 12 weeks). As linear mixed models use all available data at each time point, no missing data imputation will be performed. Age and body mass index (BMI) will be included as covariates by adding them to the regression model. The clinical classification CEAP will be included as covariate if randomization imbalance occurs. The analyses will be based on the intention-to-treat principle, including data of all randomized participants with at least one outcome measure. The significance level will be set at 95% (*p* < 0.05). All participants will be included in the analysis of the original groups following the Consolidated Standards of Reporting Trials (CONSORT) recommendations.

## Discussion

Several observational studies have reported that participants with CVI have inadequate calf-muscle pumping [14]. Calf-muscle pumping is the primary mechanism to promote blood return from the lower limbs to the heart. During exercise, the calf muscles (gastrocnemius and soleus) contract and compress the deep intramuscular veins which increases venous pressure and increases blood flow from the deep venous system to the heart. This efficacy of this mechanism depends on talocrural mobility, venous competence, and the contraction strength of the calf muscles [14].

Studies have shown the physical and QOL benefits of exercise therapy for patients with CVI. Despite positive results, this training modality is not widely used for this population. Few researchers have shown the beneficial effects of different supervised or domiciliary exercise modalities on specific parameters such as improved calf-muscle pumping [13–15], increased mean peak torque [13], improvement in disease severity [17], increased ankle-joint movement [14], and improved calf-muscle resistance [15]. The authors believe that the study results will promote preliminary evidence to help health professionals indicate, prescribe and execute supervised exercises for treating symptoms in participants with CVI.

## Trials status

Protocol version

8 July 2019 – version 1.

The first patient will be recruited in January 2020.

The last patient will be recruited in May 2020.

## Data Availability

Data-sharing is not applicable to this article as no data sets were generated or analyzed during the current study.

## Abbreviations

ABI: Ankle-brachial index
ACSM: American College of Sports Medicine
CG: Control group
CVI: Chronic venous insufficiency
PAD: Peripheral arterial disease
ROM: Range of motion
SEM: Superficial electromyography
ST6: Step test 6
TG: Treatment group
VED: Vascular echo-Doppler
